# Dermatologic immune-related adverse events associated with immune checkpoint inhibitors: A prospective real-world observational study in Switzerland

**DOI:** 10.1016/j.jdin.2026.05.023

**Published:** 2026-06-08

**Authors:** A. Trösch, C.A. Zahn, S. Cazzaniga, B.C. Özdemir, L. Feldmeyer, M. Benzaquen

**Affiliations:** a Department of Dermatology, Inselspital, Bern University Hospital, University of Bern, Switzerland; b University of Bern, Bern, Switzerland; c Centro Studi GISED, Bergamo, Italy; d Department of Medical Oncology, Inselspital, Bern University Hospital, University of Bern, Switzerland; e Department of Dermatology, University Teaching and Research Hospital of the University of Lucerne, Lucerne, Switzerland

**Keywords:** dermatitis, dermatological, epidemiology, immune-related adverse events, immune checkpoint inhibitors, immunotherapy, real-world study

*To the Editor:* Dermatologic immune-related adverse events affect 30% to 50% of patients receiving immune checkpoint inhibitors (ICIs). Yet, current management guidelines frequently rely on generic descriptors such as rash, failing to capture their true clinicopathologic diversity.^1^ Prospective data systematically integrating histopathologic evaluation remain limited.^2^

We performed a prospective observational study at the University Hospital of Bern, Switzerland (from April 2023 to October 2025). Sixty-seven adults treated with PD-1, PD-L1, or CTLA-4 inhibitors underwent standardized dermatologic evaluation. Skin biopsies and direct immunofluorescence were obtained when clinically indicated. Statistical analyses employed the Mann-Whitney U test and Fisher exact test (*P* < .05). The study received ethics committee approval.

Median age was 66 years (IQR 59-73); 67.2% were male. Melanoma (49.3%) and nonsmall-cell lung cancer (25.4%) were the most common malignancies; 89.5% had stage III-IV disease. Pembrolizumab was most frequently used (50.7%), followed by nivolumab (20.9%), and combined ipilimumab–nivolumab therapy (19.4%). Cutaneous toxicity manifested during ICI therapy in 85.1% and after discontinuation in 14.9%, consistent with delayed immune-related events.^3^ The most common final diagnoses were eczematous dermatitis (23.9%), vitiligo or leukotrichia (14.9%), lichenoid or interface dermatitis (13.4%), psoriasiform dermatoses (11.9%), bullous and erosive dermatoses (11.9%), and pruritus (11.9%) (Table I). Eczematous dermatitis exhibited significantly earlier onset (median 1.6 months, IQR 0.3-4.5; *P* = .02), potentially reflecting early barrier disruption and type 2–skewed immune activation. Notably, 90% of vitiligo cases occurred in melanoma patients, consistent with shared melanocyte-melanoma antigens and enhanced antitumor immunity.

**Table I:** Patient demographics, treatment, and dermatologic findings

Demographic characteristics	*N* = 67	%
Sex		
Male	45	67.2%
Female	22	32.8%
Age (y)		
Median, IQR	66	59.0-73.0
<65	28	41.8%
65-79	35	52.2%
≥80	4	6.0%
Tumor group		
Melanoma	33	49.3%
Non–small cell lung cancer	17	25.4%
Renal cell carcinoma	3	4.5%
Urothelial carcinoma	2	3.0%
Breast carcinoma	3	4.5%
Gastrointestinal carcinoma	3	4.5%
Nonmelanocytic skin cancer	2	3.0%
Rare/other	4	6.0%
Tumor stage		
I	2	3.0%
II	5	7.5%
III	21	31.3%
IV	39	58.2%
ICI		
Pembrolizumab	34	50.7%
Nivolumab	14	20.9%
Ipilimumab + Nivolumab	13	19.4%
Cemiplimab	2	3.0%
Durvalumab	3	4.5%
Atezolizumab	1	1.5%
Time to skin changes (mo)		
Median, IQR	3.6	1.3-8.1
since ICI started (cycles)	6	2.0-11.0
First manifestation of skin changes		
During ICI therapy	57	85.1%
After ICI therapy	10	14.9%
Histology results (*n* = 40)		
Lichenoid or interface dermatitis	13	32.5%
Granulomatous or sarcoidal	1	2.5%
Bullous	4	10.0%
Eczematous (spongiotic)	9	22.5%
Psoriasiform	6	15.0%
Vitiligo	2	5.0%
Acantholytic	1	2.5%
Neutrophilic	2	5.0%
Nonspecific or other	8	20.0%
DIF (*n* = 9)		
Positive	5	55.6%
Nonspecific	4	44.4%
Final diagnosis		
Lichenoid or interface dermatitis	9	13.4%
Psoriasiform dermatoses	8	11.9%
Eczematous (spongiotic) dermatitis	16	23.9%
Bullous and erosive dermatoses	8	11.9%
Granulomatous or sarcoidal reactions	1	1.5%
Neutrophilic dermatoses	1	1.5%
Vitiligo or leukotrichia	10	14.9%
Pruritus or pruritus sine materia	8	11.9%
Nonspecific or other dermatoses	6	9.0%

*DIF*, Direct immunofluorescence; *ICI*, immune checkpoint inhibitor.

Histopathologic evaluation, performed in 40 patients (59.7%), identified lichenoid or interface dermatitis (32.5%), spongiotic dermatitis (22.5%), and psoriasiform patterns (15.0%) as leading findings. Direct immunofluorescence confirmed bullous pemphigoid in 5 cases via linear IgG and C3 deposition along the basement membrane zone. Biopsy was essential for differentiating bullous pemphigoid from toxic epidermal necrolysis (TEN) and confirming rare diagnoses. Three life-threatening epidermal necrolysis reactions occurred: one Stevens-Johnson syndrome/TEN overlap under nivolumab, one TEN and one Stevens–Johnson–like reaction under pembrolizumab. All autoimmune bullous cases occurred exclusively with PD-1 inhibitors, consistent with existing literature.^4^ Rare histologically verified entities included sweet syndrome, Grover disease, lichen striatus, and morphea.

Comparison of PD-1 monotherapy (*n* = 50) with combined CTLA-4/PD-1 blockade (*n* = 13) revealed temporal differences (Table II). Median time to cutaneous toxicity was significantly shorter with combination therapy (0.5 vs 5.3 months; *P* < .001).^5^ Combination-treated patients more often had melanoma (84.6% vs 44.0%; *P* = .009), and stage IV disease (84.6% vs 56.0%). However, the distribution of final diagnoses did not differ significantly, suggesting that dual checkpoint blockade accelerates onset without fundamentally altering the spectrum of cutaneous toxicity.

**Table II:** Comparison of cutaneous toxicity between PD-1 monotherapy and combined CTLA-4/PD-1 blockade

	PD-1		CTLA-4 + PD-1		*P*∗
	*n* = 50	%	*n* = 13	%	
Sex					
Male	36	72.0%	6	46.2%	.1
Female	14	28.0%	7	53.8%	
Age (y)					
Median, IQR	70.5	59.0-75.0	64	52.0-65.0	.05
Tumor type					
Melanoma	22	44%	11	85%	.009
Other	28	56%	2	15%	
Tumor stage					
I-II	6	12.0%	0	0.0%	.06
III	16	32.0%	2	15.4%	
IV	28	56.0%	11	84.6%	
ICI duration (mo)†					
Median, IQR	8.5	4.9-13.3	1.3	0.7-3.1	< .001
Time to skin changes (months)†					
Median, IQR	5.3	2.0-9.4	0.5	0.3-2.1	< .001
Final diagnosis					
Lichenoid or interface dermatitis	8	16.0%	0	0.0%	.19
Psoriasiform dermatoses	6	12.0%	1	7.7%	> .99
Eczematous (spongiotic) dermatitis	10	20.0%	5	38.5%	.27
Bullous and erosive dermatoses	8	16.0%	0	0.0%	.19
Granulomatous or sarcoidal reactions	1	2.0%	0	0.0%	> .99
Neutrophilic dermatoses	1	2.0%	0	0.0%	> .99
Vitiligo or leukotrichia	6	12.0%	4	30.8%	.19
Pruritus or pruritus sine materia	6	12.0%	2	15.4%	.66
Nonspecific or other dermatoses	4	8.0%	1	7.7%	> .99

*ICI*, Immune checkpoint inhibitor.

∗ Differences across ICI therapies were investigated by using Pearson’s Χ^2^ test, or Fisher’s exact test when required, for nominal variables and Mann-Whitney U test for continuous or ordinal variables.

† Since ICI started.

In summary, dermatologic immune-related adverse events represent a heterogeneous clinicopathologic spectrum that is insufficiently captured by nonspecific descriptors such as rash. Histopathologic evaluation substantially refines diagnosis, particularly in bullous and neutrophilic dermatoses, and is critical for distinguishing severe reactions such as epidermal necrolysis. The early onset observed—especially under combination therapy—and occurrence after treatment discontinuation underscore the need for continuous dermatologic surveillance throughout and beyond ICI therapy.

## Conflicts of interest

None disclosed.
